# Transfer or tailor? Implementing a technology‐supported intervention for noncommunicable diseases across contexts

**DOI:** 10.1002/hcs2.26

**Published:** 2022-11-22

**Authors:** Thomas Gadsden, Anushka Patel, Devarsetty Praveen, Anna Palagyi

**Affiliations:** ^1^ The George Institute for Global Health University of New South Wales Sydney New South Wales Australia; ^2^ The George Institute for Global Health Hyderabad India; ^3^ Prasanna School of Public Health Manipal Academy of Higher Education Manipal Karnataka India

**Abbreviations**
CDSS: clinical decision support system
GAP: Global Action Plan
GPs: general practitioners
NCDs: noncommunicable diseases
SMART*health*
: Systematic Medical Appraisal Referral and Treatment

In May 2022, the 75th World Health Assembly recognized the need to accelerate national responses to the growing burden of noncommunicable diseases (NCDs) to achieve the goals of the Global Action Plan (GAP) for the Prevention and Control of NCDs. To facilitate this, an implementation roadmap was adopted that urges countries to prioritize the implementation of NCD interventions that are most appropriate to their specific local and regional context. The roadmap will act as an overarching guide for countries to tackle NCDs via three approaches: (i) Accelerate national responses based on local NCD epidemiology, risk factors and identified barriers and enablers; (ii) Prioritize and scale up the implementation of most impactful and feasible interventions according to the local context; and (iii) Ensure timely, reliable and sustained national data on NCD risk factors and mortality for data driven actions and to strengthen accountability [1].

With the end date of 2030 for the NCD‐GAP fast approaching, the adaptation of proven interventions is likely the most efficient and effective means by which countries can make inroads into NCD control [2]. While investment in continued research and innovation to support such adaptation processes is vital, prior knowledge and experience with the implementation of NCD interventions highlight a series of common principles that—if leveraged—can provide programs for NCD prevention and management with the strongest chance of success. In this perspective piece we share such key principles emerging from our own experience implementing a multifaceted NCD management intervention—SMART*health* (Systematic Medical Appraisal Referral and Treatment)—across different country contexts.

SMART*health* is a technology‐supported, multifaceted primary health care intervention aimed at improving the provision of guideline‐based assessment and prevention or management of common NCDs. A central common component of the SMART*health* intervention is a clinical decision support system (CDSS) with context‐defined variations in disease focus and approach to health system integration, including workforce strategy [3] (Figure 1).

The intervention was first piloted in Australia as a CDSS embedded in existing patient management information systems and delivered by general practitioners (GPs) [4]. Encouraged by positive outcomes (e.g., improved cardiovascular risk factor screening, namely blood pressure [BP] recording and up‐titration of cardiovascular preventive drugs), the CDSS was adapted to the Indian primary health care setting by expanding the platform to include a mobile application that can be used by frontline health workers within rural communities to support early identification, referral, and management for cardiovascular disease. Though the platform was found to be acceptable by the community and health professionals across test sites in rural India, no clear evidence of clinical benefit emerged from a cluster randomized trial (e.g., no difference in achieving BP targets or receiving BP‐lowering medication) [5, 6]. Conversely, a subsequent trial in similar primary health care settings in rural Indonesia was strongly positive (e.g., higher use of BP‐lowering medication and lower BP levels in those receiving the intervention), leading to the intervention being adopted by the local government authority for scale‐up [7]. To further evaluate variation in implementation outcomes, pilots of the SMART*health* intervention are currently ongoing in China and Thailand [8].

Our experience adapting this complex intervention across five diverse health systems (Australia, India, Indonesia, China, and Thailand) has highlighted four common principles that underpinned implementation in each setting: [1] Assessment of readiness of the new setting for implementation of the intervention; [2] Integration of the intervention with existing policies, systems and infrastructure; [3] Leveraging (and adequately training/supporting) an existing health workforce; and [4] Harnessing community engagement and local leadership for ownership and sustainability.

1. Assessment of readiness of the new setting for implementation of the intervention
  When considering the adaptation of a proven intervention to a new setting, it is critical to understand which intervention components require high implementation fidelity and the readiness of the new setting to accommodate these. Adaptation of the SMART*health* intervention for each setting was preceded by careful assessment of the existing health policy landscape, disease specific guidelines, health care infrastructure and health workforce organization. For example, when implementation was planned in Indonesia following lessons learned in India (where there was less focus on codesign), there were concerted efforts to codesign and continuously improve implementation strategies to ensure best fit for context. This included a systematic analysis of the policy landscape relating to essential medicines supply to plan medication prescription and distribution at the community level, and also an assessment of community awareness of NCDs that led to the integration of an initial health awareness component in the intervention package [7]. A similar barrier analysis was also initially conducted in China to understand contextual constraints on the provision of best practice care [8]. Comparatively, an opportunity to implement the intervention in Sri Lanka was not pursued following an initial health system assessment because of an inherent separation of primary curative care and preventive services, lack of a relevant community workforce and an evolving IT infrastructure.
2. Integration with existing policies, systems and infrastructure
  Based on these assessments, intervention components were integrated to the local context to maximize fidelity while ensuring minimal changes to the existing healthcare workflow. In Australia, the CDSS was designed to interface with existing patient management information systems used by GPs [9]. This did not change the clinical management workflow but provided additional information (regarding patient's NCD risk status) to support GPs in their patient management. Similarly, in Indonesia the SMART*health* mobile application was designed to interface with and send data to the local primary health care electronic medical record database (ePuskesmas). In India, a process evaluation of the initial intervention trial found that the effect of SMART*health* was likely diluted by other on‐going initiatives, suggesting the intervention was not sufficiently integrated into existing policies and systems, receiving limited “buy in” from local healthcare system stakeholders [10].
3. Leveraging (and adequately training/supporting) an existing health workforce
  One of the core intervention components for high implementation fidelity which was considered in our initial assessment of system readiness was the presence of a health workforce with capacity to deliver the intervention. In India, Indonesia and Thailand the presence of an existing community health care workforce delivering care through household visits provided task‐sharing opportunities, avoiding the need to develop an entirely new cadre of worker. Patient screening and follow‐up for the intervention was conducted by Kaders in Indonesia, Accredited Social Health Activists (ASHAs) in India and, currently, by Village Health Volunteers in Thailand. In China, this role was fulfilled by Family Health Promoters, voluntary family members who have received basic training in chronic disease management with responsibility for maintaining the health of their family [8]. In contrast, in the Australian context, local GPs used the CDSS as per the norms of clinical management in that country.
  However, relying on an existing health workforce to deliver a new intervention has the potential to perpetuate prevailing challenges—including those associated with capacity, scope of practice and incentives. To ensure the SMART*health* mobile application appropriately met the needs and capabilities of frontline health workers in India, an “agile development methodology” was adopted [3, 11]. This included iterative development, testing with ASHAs who had little experience using information and communication technology, and refinement based on their feedback. In all SMART*health* implementation settings additional training was also provided to frontline health workers to build their capacity to manage NCD conditions and, where appropriate, higher level health workers (such as nurses) were engaged to provide monitoring and additional training and support. While this level of investment may not be feasible in all settings, the principle of adapting interventions in line with the capacity of the local workforce remains relevant.
4. Harnessing community engagement and local leadership for ownership and sustainability
  A key reason for the success of the 2‐year trial of the SMART*health* intervention in Indonesia was the high level of investment from local healthcare system stakeholders. This was facilitated by a local Expert Advisory Group led by champions from within the District Health Authority, together with representatives from the national health insurance agency and health care personnel. Before the initiation of the program at the village level, the District Health Authority visited village elders and representatives to promote the importance of NCD screening. Additionally, senior district government officials created a supportive policy and logistical environment for the implementation of the intervention by committing to improve existing supply chain issues by investing in the procurement of essential cardiovascular disease medicines to meet the likely demand. Local investment has continued in the district‐wide scale up of the intervention, which is government led, to 390 villages and a targeted population of 1 million adult residents.
  Implementation of SMART*health* in countries across a spectrum of health system contexts has provided important lessons emerging from both successes and failures in field trials. For policymakers seeking to accelerate their national NCD response, our experience suggests that contextual adaptation of proven interventions is an important option in their toolbox. However, substantial time and investment is required. The lessons learned and shared here are not intended to be definitive and exclusive, but to improve opportunities for success by ensuring that the adaptation of interventions is appropriate, integrated and accepted in their new implementation setting. However, we recognize that a limitation of these learnings is they have been accumulated by “researchers looking in” and based on pilot implementation and evaluation. The insights of health system administrators, policymakers, providers and community members are also critically needed, and are something that we hope to learn during SMART*health* scale up in Indonesia.

**Figure 1:**
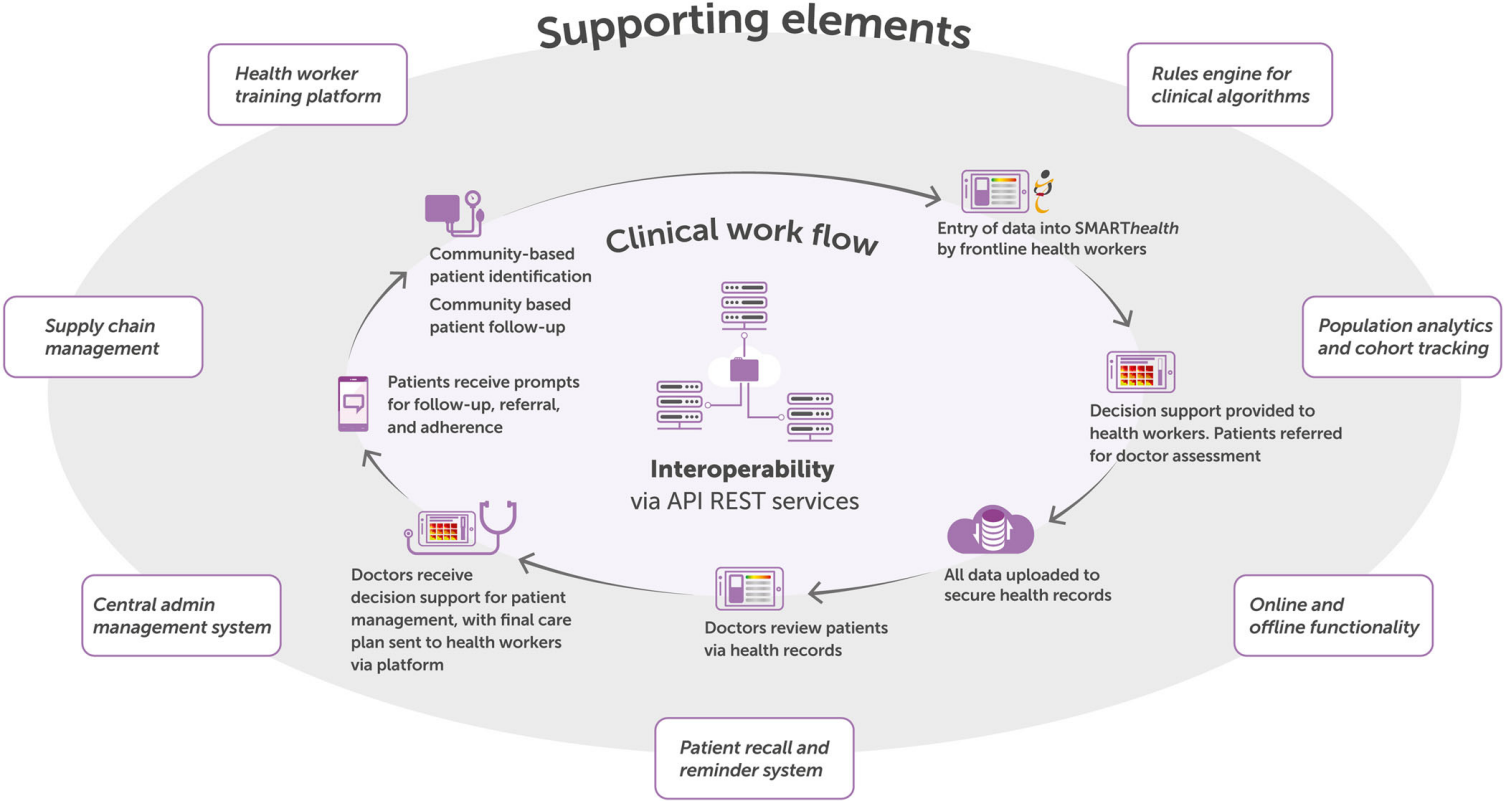
SMART*health* intervention cycle

## KEYWORDS

context, health system interventions, noncommunicable diseases

## AUTHOR CONTRIBUTIONS

**Thomas Gadsden**: Writing – original draft (lead); writing – review and editing (lead). **Anushka Patel**: Conceptualization (equal); project administration (lead); writing – review and editing (equal). **Devarsetty Praveen**: Writing – review and editing (supporting). **Anna Palagyi**: Conceptualization (equal); writing – original draft (equal); writing – review and editing (equal).

## CONFLICT OF INTEREST

The author declares no conflict of interest.

## ETHICS STATEMENT

None declared.

## INFORMED CONSENT

None.

## Data Availability

Data sharing is not applicable to this article as no new data were created or analyzed in this study.
